# Cesarean section in sub-Saharan Africa

**DOI:** 10.1186/s40748-016-0033-x

**Published:** 2016-07-08

**Authors:** Margo S. Harrison, Robert L. Goldenberg

**Affiliations:** Columbia University Medical Center, 622 W 168th St, PH16, New York, NY 10032 USA

**Keywords:** Cesarean section, Low- and middle-income countries, Trial of labor after cesarean section, Vaginal birth after cesarean section, Epidemiology, Sub-Saharan Africa

## Abstract

Cesarean section is an essential maternal healthcare service. Its role in labor and delivery care in low- and middle-income countries is complex; in many low-resource settings it is underutilized in the most needy of populations and overused by the less needy, without clear methods to ensure that universal access is available. Additionally, even if universal access were available, it is not evident that these countries would have the capacity or the finances to appropriate meet demand for the procedure, or that patients would want to utilize the care. This review summarizes the literature and illustrates the complicated relationship that cesarean section, which is rapidly on the rise around the world, has with individuals, communities, and nations in sub-Saharan Africa.

## Background

At the top of the World Health Organization’s (WHO) agenda regarding maternal mortality is improving the availability, accessibility, quality, and use of services for the management and treatment of complications of pregnancy, labor, and delivery [1]. As such, the WHO has defined a concept of emergency obstetric care services (EmOC) which requires, for a basic level of services, that an institution be able to provide parenteral antibiotics, uterotonic drugs, intravenous magnesium sulfate, and have providers who can manually remove a placenta, remove retained products of conception, perform assisted vaginal delivery, and perform basic neonatal resuscitation [1]. Comprehensive care, referred to as comprehensive EmOC, requires the additional ability of a health service organization to perform cesarean section (CS) and administer a blood transfusion [1]. It should be noted that CS is considered essential treatment for antepartum hemorrhage, prolonged or obstructed labor, pre-eclampsia or eclampsia, and intrapartum fetal distress [1]. It is in these situations that CS can avert major obstetric complications that lead to maternal, neonatal, and/or fetal death.

## Epidemiology

**Fig. 1:**
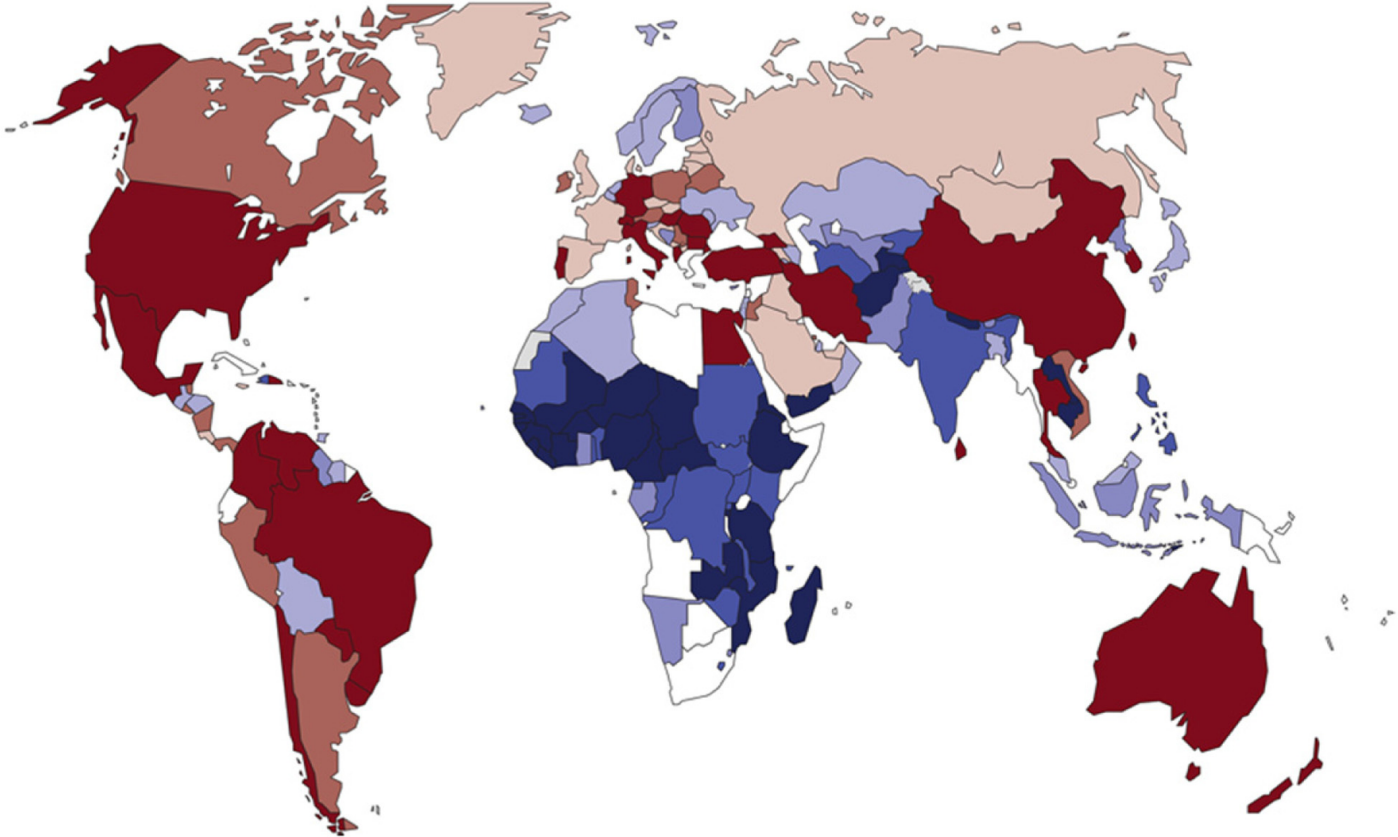
Latest available data on cesarean section rates by country (not earlier than 2005)

Per WHO, it has been established that CS is an essential treatment in pregnancy and is recommended at a rate of 5–15 % of all births [1]. Epidemiologic studies have shown that in high-income (HIC) and some low- and middle-income countries (LMIC) alike, CS is being provided at higher, and sometimes much higher, rates than recommended. A recent WHO publication reports that between 1990 and 2014 the global average CS rate increased from 12.4 to 18.6 % with rates ranging, depending on region, between 6 and 27.2 %, and rising at an average rate of 4.4 % per year [2]. The lowest rates were found in Africa (7.3 %), followed by Asia (19.2 %), Europe (25 %), Oceania (31.1 %), and North America (32.3 %), with Latin America and the Caribbean having the highest rates at 40.5 % [2]. Figure 1, from this article, shows the latest available data on CS rates by country [2]. The paper also shows a graphic on global and regional trends in CS from 1990 to 2014 (Table 1). While all the other regions showed an increase in CS, there was a small, but real increase in the CS rates in sub-Saharan Africa (SSA) over that time period, as well.

**Table 1:** Caesarean delivery rates among richer and poorer women in urban and rural areas, southern Asia and sub-Saharan Africa, 2003-2011

Country	Caesarean delivery rate^a^				Absolute difference^b^ (95 % CI)
	Rural poorer	Rural richer	Urban poorer	Urban richer	
Southern Asia					
Bangladesh	2.29	11.52	1.32	20.37	10.19 (7.73 to 12.65)
India	3.59	15.23	5.99	21.75	9.25 (7.44 to 11.05)
Nepal	1.51	7.03	4.40	17.24	2.63 (−1.97 to 7.23)
Pakistan	2.00	10.50	1.65	14.97	8.85 (6.53 to 11.18)
Western and central Africa					
Benin	1.76	3.00	1.78	7.23	1.22 (0.26 to 2.19)
Burkina Faso	0.76	1.48	3.23	6.11	−1.75 (−3.35 to −0.16)
Cameroon	0.51	1.79	1.75	4.11	0.04 (−1.46 to 1.53)
Chad	0.18	0.33	0.00	1.53	0.33 (−0.19 to 0.84)
Cote d’Ivoire	1.39	7.17	4.04	7.30	3.13 (−9.19 to 15.44)
Ghana	3.22	9.50	4.49	10.80	5.01 (−0.27 to 10.30)
Guinea	0.38	1.77	0.71	4.76	1.06 (−0.71 to 2.83)
Mali	0.27	0.69	1.41	2.39	−0.72 (−2.23 to 0.79)
Niger	0.34	0.37	1.93	4.60	−1.57 (−5.66 to 2.53)
Nigeria	0.35	2.49	0.67	4.05	1.82 (0.99 to 2.66)
Senegal	1.37	2.89	2.62	9.77	0.28 (−2.15 to 2.70)
Eastern and southern Africa					
Ethiopia	0.39	0.63	1.17	8.38	−0.54 (−2.20 to 1.12)
Kenya	3.21	9.41	2.69	11.16	6.72 (3.02 to 10.43)
Lesotho	3.35	7.71	8.23	11.50	−0.52 (−12.36 to 11.32)
Madagascar	0.32	2.08	1.62	5.89	0.46 (−1.87 to 2.80)
Malawi	3.23	4.96	2.94	8.44	2.02 (−1.31 to 5.34)
Mozambique	0.32	1.14	0.94	5.99	0.20 (−1.10 to 1.51)
Rwanda	5.01	6.70	7.51	17.53	−0.81 (−5.72 to 4.09)
Uganda	2.76	5.91	7.55	13.96	−1.63 (−8.02 to 4.76)
United Republic of Tanzania	2.30	4.55	0.95	9.96	3.60 (1.70 to 5.51)
Zambia	1.22	3.25	0.00	5.90	3.25 (1.79 to 4.70)
Zimbabwe	2.88	3.68	2.67	8.19	1.01 (−2.72 to 4.74)

*CI* confidence interval

^a^ Caesarean delivery rates are expressed as percentages of the deliveries that ended in a live birth, excluding all but the last born of the neonates delivered in each multiple birth. They take into account sampling weights. The corresponding CIs take into account sampling weights, clustering and stratification. Women who lived in households that had wealth indices that fell above the national median value were considered to be “richer”, whereas other women were categorized as “poorer”

^b^ The caesarean delivery rate for the rural richer minus the corresponding rate for the urban poorer

Note: The data presented come from the most recently published Demographic and Health Survey in each country

## Why cesarean section is on the rise in SSA

With CS rates increasing globally, it is of the upmost importance to discern why this intervention is being provided more frequently, and at such rapidly increasing rates in many settings, albeit slower rates in SSA. CS is considered an appropriate intervention for antepartum hemorrhage, prolonged or obstructed labor, pre-eclampsia or eclampsia, and intrapartum fetal distress. As such, many researchers have tried to determine for what indications CS are actually being performed in SSA, to find out if the procedure is being performed unnecessarily, and which factors are contributing to the increasing number of CS.

### Audits

Many institutions have published audits of their experience to determine why CS were performed and for what indications. A Tanzanian study which included chart review and staff interviews found that suboptimal management occurred in most cases and that there was a lack of awareness and use of evidence-based guidelines, leading to unnecessary CS [3–5]. Similar conclusions were drawn by researchers in Burkino Faso and Ethiopia, who also found that audits of the appropriateness of cesarean section were essential along with the development of clinical management protocols to reduce the rate of unnecessary CS [6, 7]. In terms of actual indications cited, a Medicins sans Frontiers multi-country analysis conducted in sub-Saharan Africa, reports that the indications for CS were obstructed labor (31 %), malpresentation (18 %), prior cesarean section (14 %), fetal distress (10 %), uterine rupture (9 %), and antepartum hemorrhage (8 %); no comment was made regarding the appropriateness of the indications or about whether a certain percentage of these CS were unindicated [8].

### Risk factors

Some studies have identified socio-demographic risk factors that contribute to an increased risk of CS in certain populations. A study from Ethiopia noted that women with a secondary or higher level of education were nearly two times more likely to undergo CS than women with no or only a primary education, that ‘rich’ women were significantly more likely to receive CS than women from ‘poor’ or ‘middle’ income households, and that women who delivered in private institutions were twice as likely to be delivered by CS than their counterparts who delivered in public institutions [9]. The finding that richer women are more likely than poorer women to receive CS is noted consistently across studies. A study on trends in CS by country and wealth quintile in southern Asia and sub-Saharan Africa found that among the poorest 20 % of the population, CS accounted for less than 2 % of deliveries among a majority of countries studied, and in some countries the rate was less than 1 % in the poorest 80 % of the population [10]. Table 1 illustrates these findings with a focus on rich and poor women who live in urban versus rural settings [10]. Similarly, a study from Tanzania showed that large variations in CS levels were observed between different socio-demographic groups, possibly reflecting inequitable access to services [11].

In terms of non-financial factors associated with CS, an epidemiologic study of 86,505 women who delivered at referral hospitals in Senegal and Mali determined independent individual factors that were associated with CS [12]. The authors found a rate of intrapartum CS of 14 %, an emergent CS (which was not clearly defined) rate of 3 %, and that 2 % of CS were elective [12]. Notably, the presence of obstetricians and/or medical anesthetists was associated with an increased prevalence of elective CS [12]. For all types of CS, the main maternal risk factors were prior CS, referral from another facility, suspected cephalopelvic disproportion, vaginal bleeding near term, hypertensive disorders, previous CS, and premature rupture of membranes [12].

### Induction of labor and CS rates

In many HIC, induction of labor (IOL) has been debated as a risk factor for CS. Therefore, understanding the role that IOL plays in the increasing rate of CS in LMIC is in order. An analysis of the WHO Global Survey on Maternal and Neonatal health dataset was used to try to understand the patterns and outcomes of IOL in Africa and Asia [13]. The results suggested that IOL was generally less common in LMIC than in HIC, that prostaglandin use was rare, and oxytocin was the most common method utilized. IOL accounted for 4.4 and 12.1 % of deliveries in Africa and Asia, respectively [13]. The success rates were generally over 80 %. Medically indicated inductions in both regions were associated with an Apgar score of <7 at 5 min, low birthweight, NICU admission, and fresh stillbirth [13]. The analysis concluded that despite the fact that one-third of elective inductions were reportedly performed at less than 39 weeks gestational age, the risks of maternal, neonatal, and fetal mortalities were not elevated [13]. While this analysis suggests that IOL does not increase the rate of CS and may actually result in the converse, the authors of this analysis warn that despite growing use of elective labor induction globally, questions remain about the safety, risks, benefits, and cost-effectiveness. They assert that IOL should only be performed in the context of informed consent, access to comprehensive EmOC, and appropriate maternal and fetal monitoring and supervision [14].

## Access to and capacity of SSA countries to provide cesarean section

Inadequate finances, services, equipment, and medications often characterize the healthcare systems in SSA. With global CS rates rising, but inconsistently so among and within countries, and with evidence suggesting that CS may be utilized predominantly by wealthy urbanites, there is concern about the access to and capacity of SSA to provide CS to those women for whom it is indicated, in all settings.

### Access

The greatest burden of maternal mortality falls on LMIC, accounting for about 99 % of the estimated 300,000 deaths that occur per year [15]. One evidence-based intervention for reducing maternal morbidity and mortality is promoting delivery in a facility by a skilled birth attendant, which includes access to CS [16]. However, challenges affect access to CS in SSA. A review article that evaluated studies from 16 countries found that major barriers to achieving higher rates of CS (the average rate was 3.6 %) were poverty and limited access to healthcare services, as well as a shortage of healthcare providers [17]. In Tanzania, a study found that women referred for delivery in a facility had higher rates of CS and poorer neonatal outcomes, suggesting that the formal referral system was identifying high-risk pregnancies, but that there was a need to target women for earlier professional intrapartum care [18]. Similarly, a study from Kenya found that rural women face higher risks of dying during pregnancy and childbirth, and improving access to life-saving interventions in underserved areas should be a priority [19].

Some interventions have been tested to improve access to EmOC in low-resource settings. In South Sudan, an ambulance referral system was put in place and patients were given transport and hospital care free of charge [20]. This resulted in an increased CS rate (4.9 %), with 99.1 % of women that had obstetric indications for EmOC being treated in the hospital [20]. Similarly, in Uganda, a 24-h ambulance service was implemented to transport laboring women to the hospital, which resulted in an increased CS rate from 0.57 to 1.21 %. The CS rate remained stable in the non-intervention, control district where transportation was not offered [21]. The authors also reported that hospital deliveries increased by over 50 % per year with a non-significant decrease in stillbirths during the intervention period [21]. A study in Mali reinforced that access not only relies on transport, but also proximity to a facility [22]. This study showed that a travel time of four or more hours was significantly associated with increased in-hospital maternal mortality, and concluded that improving spatial access to EmOC will help women arrive at the facility in time to be treated effectively [22]. For comparison, a multi-country trial of a package of interventions including community mobilization, transportation, community birth attendant training, and facility staff training in obstetric and neonatal emergencies was undertaken to determine what, if any, effect the intervention would have on pregnancy outcomes [23]. This cluster-randomized comprehensive, large-scale, multi-sector intervention did not result in detectable impacts on perinatal mortality or birthweight, which were the primary outcomes of interest [23]. The authors conclude that a lack of quality hospital care was the likely reason the intervention had little impact [23].

Finally, in a synthesis of qualitative evidence from 34 countries as to which characteristics facilitate or represent a barrier to facility-based delivery for women in LMIC, the authors concluded that, accessing facility-based delivery care involves input from many actors and is influenced by myriad physical and sociocultural factors [24]. They found that women often yearn for the supportive attendance, privacy, and familiar practices that they experience while delivering at home and that prevents them from attending a facility [24]. Additionally, they found that the inaccessibility of facilities due to geographical barriers and the high costs of facility-based delivery are critical barriers as well [24]. They concluded that government policies, insurance schemes, and other public health programs often fail to effectively mitigate these physical barriers due to poor implementation and that mistreatment, abuse, and neglect by health workers have fostered dissatisfaction, distrust, and avoidance of facility-based delivery care in many contexts [24]. This article suggests that access to care in SSA, and LMIC in general, may be more complex than the “three-delays” model that previously described complications related to facility delivery [25]. The three delays model is one introduced by authors Thaddeus and Maine, which suggests that delays in delivering in a healthcare facility can occur at three different levels: (1) delay in decision to seek care, (2) delay in reaching the appropriate facility and (3) delay in receiving adequate care in the facility [25].

### Capacity

If availability of facilities were not a problem and every woman living in a SSA country decided to deliver in such a setting, the question would arise as to whether or not the countries have the capacity to provide emergency obstetrical care for their populations. The WHO has asserted that one institution able to provide comprehensive EmOC, which requires the ability to perform CS, should be available per 500,000 people [1]. A few large-scale studies have been performed on LMIC capacity to perform CS. The first describes staffing and availability of equipment, medications, and procedures at facilities in African, Asian, and Latin American sites with high maternal and perinatal mortality [26]. The authors found that only 20 % of hospitals in Africa had full time physicians, only 70 % of hospitals in Africa and Asia had performed a CS in the prior 6 months, and blood was unavailable in 80 % of African and Asian hospitals [26]. Similarly, another study assessing CS availability in 26 LMIC found that while 73.8 % of facilities reported the ability to perform CS; 47.3 % of these did not report the presence of any anesthesia provider, and 17.9 % reported no obstetrician/gynecologist or surgical provider was available [27].

Studies focused purely on capacity in SSA are also present in the literature. A study performed in Burkino Faso found that the indicator of one comprehensive EmOC institution per 500,000 population was not achieved within the country [28]. The analysis found that only 27.8 % of hospitals at the time of the study could continuously offer CS and blood transfusion services [28]. While a similar study in Kenya found that the ratio of comprehensive EmOC facilities per 500,000 was appropriate, none of the facilities met the true definition of comprehensive EmOC as operative vaginal delivery was not offered [29]. The study also showed a CS rate of <5 % in rural areas as compared to urban areas where CS rates reached about 7 % [29]. Data from southwest Ethiopia reported a lack of comprehensive EmOC facilities to meet the needs of its large population, and the utilization of existing facilities for deliveries was also low at less than 2 % in remote districts and 6.6 % overall [30]. Whether determined by large database analyses or from single-country surveys, the data suggest that in LMIC, particularly in sub-Saharan Africa, CS capacity is not sufficient to meet WHO standards.

## Outcomes of cesarean section in SSA

The experience from HIC illustrates that adverse events do occur in the setting of cesarean delivery, such as increased blood loss, damage to pelvic organs, and increased risk of thromboembolism, among other complications, but that CS can prevent stillbirth and maternal morbidity and mortality related to pregnancy complications [31]. It would be naïve to suggest that the experience in SSA regarding CS would be different in terms of incurring complications, but the concern is that outcomes may in fact be worse, due to resource constraints. The data show that this concern is not unfounded.

### Outcomes

CS is a major abdominal surgery. It often occurs under stressful conditions as it is indicated in the setting of hemorrhage, fetal distress, hypertensive disease, and cephalopelvic disproportion, and in LMIC, is often performed by underqualified, poorly trained personnel with little surgical experience. As such, it is not surprising that despite it being performed in generally high-risk patients who have an increased baseline risk of adverse outcomes, CS can exacerbate those outcomes even further.

The South African experience informs this finding. An analysis evaluating maternal deaths in the setting of CS found that the risk of a woman dying after CS was almost three times the risk of maternal death after vaginal delivery [32]. Hemorrhage was a leading cause of death, resulting in a rate of 5.5 deaths per 10,000 CS performed [32]. As a final cause of death, embolism was 4.5 times more likely after CS than VD, and hypovolemic shock 4.8 times more likely [32]. CS case fatality rates ranged from 10.1 deaths per 10,000 CS to 31.9 [32]. When the CS rate in a facility was compared to the case fatality rate, a negative correlation was found, meaning that in areas with a lower rate of CS, there was a higher case fatality rate [32]. Overall, the study found that specific problems related to death after CS included bleeding during or after CS, pre-eclampsia and eclampsia, anesthesia-related deaths, pregnancy-related sepsis, acute vascular collapse, and embolism [32].

A WHO analysis considered “severe maternal outcome”, which includes a maternal near miss or maternal death, by delivery method [33]. The study showed that in 314,623 women in 29 African, Asian, Latin American, and Middle Eastern countries, 28.6 % were delivered by CS [33]. Of women delivered by CS, 62.5 % experienced a severe maternal outcome, which was significantly different than the 37.5 % of vaginal deliveries where a severe maternal outcome was reported [32]. The results also showed that about twice the percentage of women who had a severe maternal outcome had greater than one prior CS as compared with women who did not, suggesting that tertiary or higher CS are associated with adverse maternal outcomes [33]. The percentage of women who had a CS with no labor was three times more prevalent in the group experiencing a severe maternal outcome as compared to those who did not, suggesting that non-laboring CS were more common in women with worse pregnancy outcomes [33]. Of all patients included in the study, 26.7 % of the severe maternal outcomes were due to postpartum hemorrhage, and 25.9 % were due to hypertensive disease [33].

Another large study including 78,166 patients from Senegal and Mali also looked at maternal and perinatal outcomes by delivery method [34]. A small percentage of these women (2.2 %) had a pre-labor CS, which was associated with a significant reduction in stillbirth and neonatal mortality at <24 h of life, as compared to a trial of labor [34]. However, for those CS that were performed intrapartum (12.5 %), there was a higher risk of maternal mortality and morbidity, as well as increased risk of neonatal death at > 24 h of life [34]. This study illustrates that CS is associated with some improved outcomes, but may contribute to the exacerbation of others.

### Quality

It is difficult to determine whether a patient’s antepartum and intrapartum course prior to CS is responsible for the patient’s morbidity and mortality, or if the CS itself is responsible for some component of the adverse outcomes. Lack of access to CS can result in hemorrhage, sepsis, and urogenital fistula as well as perinatal asphyxia and stillbirth. Access to a poor quality CS, however, can result in maternal and neonatal morbidity affecting not only the incident pregnancies, but future ones as well. The WHO analysis presented in the prior section concluded that to reduce maternal mortality requires improvements in the *quality* of maternal health care [33].

Certain indicators of quality can be measured in relationship to CS. For example, incidence of surgical site infection can be monitored as a quality indicator, especially since administration of prophylactic antibiotics can reduce the incidence of wound infection. A multi-country study from sub-Saharan Africa prospectively collected data on surgical site infections in women undergoing CS [35]. The authors found that the rate of infection was 7.3 % with 93 % of infections being superficial, which is generally consistent with findings from HIC, suggesting that CS can be performed in LMIC with a low incidence of infection, and the marker can be used as a proxy for quality [36, 37]. Similarly, administration of prophylactic antibiotics itself can also be considered a quality indicator in CS. For example, a study in Tanzania showed that when prevention of CS-related infection was evaluated using administration of prophylactic antibiotics as an indicator of quality of care, only 66 % of patients received medication [37].

Another extremely important indicator of surgical quality is urogenital fistula and genitourinary injury during CS. Historically, urogenital fistula was mostly associated with neglected prolonged or obstructed labor that resulted in devascularization and necrosis of pelvic tissues. With the rise of CS, it has become apparent that poor surgical technique can also lead to fistulae [38]. When the bladder and/or ureters are damaged during surgery, neglect or lack of recognition of the injury can lead to the formation of fistulous tracts that epithelialize over time and result in urogenital fistula. Thus, surgically derived urogenital fistula serves as a quality indicator of surgical performance. While certain types of fistulae are more common in the setting of obstructed labor versus CS, both labor and surgery-related fistulae are indicators of adverse pregnancy outcomes, the former from a lack of access to appropriate care, and the latter from lack of access to quality care [38].

A study performed in the Democratic Republic of Congo reviewed fistulae believed to be the result of poor obstetric technique [38]. The authors, from a retrospective review of hospital records, found that 40 % of women with fistulae had had a CS. They attributed 24 % of these fistulae to their CS [38]. Interestingly, the analysis showed that the odds of having a surviving infant after a CS that resulted in fistula was three times higher than in those pregnancies where the fistula resulted after vaginal delivery [38]. The analysis also showed that cervical involvement was more common with fistulae after CS, that these fistulae were complicated by less fibrosis, and that there was a decreased interval to treatment in the CS group [38]. Fistula formation is an indicator that should be collected regularly as an outcome of all CS to monitor quality of care.

### Surgical standards

One way to improve quality of care is to implement surgical standards. When it comes to surgical standards for CS, research has not shown that certain techniques improve outcomes more than others. A large trial recruited over 15,000 women in five LMIC to study the following five interventions: (1) blunt versus sharp abdominal entry; (2) exteriorization of the uterus for repair versus intra-abdominal repair; (3) single-layer versus double-layer closure of the uterus; (4) closure versus non-closure of the peritoneum; and (5) chromic catgut versus polyglactin-910 for uterine repair [38]. There were no statistically significant differences within any of the intervention pairs [39]. Of note, however, the study did find that 26 of 144 adverse events that were reported were likely attributable to the CS itself, suggesting that an important percentage of complications come from the procedure itself [39].

There are only two surgical standards that the WHO has published for CS, and they are that oxytocin (IV or IM) is the recommended uterotonic drug for the prevention of post-partum hemorrhage in CS, and that cord traction is the recommended method for the removal of the placenta during CS [40]. There is clearly a need for improved guidelines on CS standard of care on a global scale.

## Cost of cesarean section in SSA

If facility-based birth was accessible to all women in low-resource settings and the healthcare systems could accommodate the provision of all indicated CS, the next logical concern would be, in financially strained economies like those in LMIC, is the cost of providing this service manageable. And what, exactly, is the cost? The WHO sought to answer a similar question by determining what it would cost to end preventable maternal, fetal, and neonatal deaths with available healthcare interventions by providing facility-based care [41]. By modeling the effect and cost of facility-based care for all women in the 75 most high-burden countries, the authors found that it would cost US $4.5 billion to prevent 113,000 maternal deaths, 531,000 stillbirths, and 1.325 million neonatal deaths [41]. Of note, they also found that increased coverage and quality of preconception, antenatal, intrapartum, and postpartum interventions could avert 71 % of neonatal deaths, 33 % of stillbirths, and 54 % of maternal deaths, which amounts to US$1928 per life saved, but is not CS-specific [41]. While these data help to give a broad perspective of the cost of essential care for women during and after pregnancy, it does not get into the logistics of the actual cost to countries, communities, and families at the country level.

### Economics

A study in Mali evaluated the cost of emergency obstetrical care to the individual and her family. The study found that despite a fee exemption policy for CS, the women and their families still incurred an expense of US $152. This amount was a catastrophic expenditure in up to 53.5 % of households, resulted in a decrease in food expenditure in 44.6 % of families, and led to remaining debt in 23.2 % of the families 2 years after the surgery [42]. Another study from Mali found that 5 years after implementation of the fee exemption policy for CS, wealthier women made up a disproportionate share of those receiving free CS, accounting for 59 % of procedures, while the women from the poorest two quintiles made up 28 % of the CS volume [43]. This suggests that removing financial barriers to CS may not resolve access issues altogether [43]. A follow-up study performed 9 years after the same policy change in Mali to evaluate the impact on CS rates found similar results; the CS rate for urban women went from 1.7 to 5.7 % while only increasing from 0.4 to 1 % for women living in villages [44].

While removing user fees does not solve all problems, it does appear to increase utilization. Data from Demographic and Health Surveys conducted in ten SSA countries found that the policy change was consistent with an increase of 3.1 facility deliveries per 100 live births, which corresponds to a 5 % increase in facility deliveries, with no corresponding increase in the rate of CS [45]. In Sudan, the free care policy applied only to CS and not to vaginal delivery, but utilization of services still increased 14 %; authors noted, however, that this policy was implemented without the resources to support it, which still led to a significant portion of the cost being born by the woman and her family [46].

A study performed in eastern Uganda documented the actual costs to the government when a voucher system was implemented to cover maternal health services and transportation [47]. The cost breakdown was 33.5 % spent on transportation of 39,348 women (US$4.6 on average to and from the facility), 29.2 % spent on strengthening the healthcare system, and maternal healthcare costs amounted to 18.2 % (13,283 women were delivered during the study), with other costs attributed to sensitization, mobilization, and administration [47]. CS cost was US$28 in a public facility and US$52 in a private setting [47]. Overall, the cost per delivery was US$23.9.

Conversely, many studies suggest that CS is a cost-effective intervention. In the Democratic Republic of Congo, a study found that with a CS rate of 9.2 %, with each procedure costing US$144, the intervention was cost-effective [48]. An analysis performed at a district hospital in Zambia also found that CS was cost-effective both in emergent (US$7.42) and elective (US$20.50) circumstances, per disability-adjusted life year gained [49]. Similarly, a study in 64 LMIC found that CS was cost-effective at a price of US$18-3,462 per disability-adjusted life year gained [50]. Overall, the data seems to support that while the cost of providing maternity care throughout the antenatal, intrapartum, and postpartum setting is not negligible, it is cost-effective.

## Long-term impact of rising cesarean section rate

Quality and short-term outcomes of CS have an immediate impact on the lives of women, their newborns, and their families. The rising rate of CS also has immediate consequences for facilities and countries in terms of volume and cost. What often gets less attention are the longer-term outcomes of CS and how it affects a woman’s future childbearing, and the resources her future pregnancies will demand from the healthcare system.

### Trial of labor after cesarean section

An editorial on safety concerns over planned vaginal birth after cesarean section in SSA brings to light the main issues [51]. The authors assert that trial of labor after cesarean section (TOLAC), also called planned vaginal birth after cesarean section (VBAC) is not as safe as it was once purported to be, and that it might be even more dangerous in LMIC given the scarcity of essential resources. They believe that the major maternal complications (uterine rupture, hysterectomy, thromboembolism, hemorrhage, transfusion, visceral injury, and maternal death) outweigh the risks of repeat CS (greater risk of severe hemorrhage requiring blood transfusion and postpartum endometritis) [51]. The authors raise the concern that the commonly quoted universal success rate is based on the vaginal delivery itself, and does not take into account ensuing complications, as facilities in HIC can manage these adverse outcomes without a significant burden [51]. They also point out that the recommended monitoring methods and safety precautions such as reviewing prior surgical records and having full-time access to a blood bank and operating room for CS are not commonly available in LMIC [51]. The authors conclude that elective repeat CS (ERCS) should be offered over VBAC, and efforts should be made to improve the safety of the procedure and the availability of operative vaginal delivery [51].

While the editorial makes many excellent assertions, the pregnancy after a CS is not the only pregnancy or future fertility to be considered. The woman, especially if she lives in a LMIC with a high pregnancy and fertility rate, may go on to have a number of future pregnancies, and morbidity and mortality increases significantly with each additional ERCS. These complications could potentially be averted by a VBAC in the pregnancy following her primary CS. This point is supported by a study in Nigeria that found that higher order CS (defined as > 3 prior CS) were more likely to have postpartum hemorrhage, have longer operating times, and receive a blood transfusion than women with 3 or fewer CS [52].

A 4-year prospective observational study was conducted in Senegal and Mali to observe maternal and perinatal outcomes between women undergoing a TOLAC after one prior CS versus those who experienced an ERCS [52]. The study found that the risk of a maternal complication (uterine rupture, postpartum hemorrhage, infection/sepsis, transfusion, hysterectomy, or maternal death) and perinatal mortality were significantly higher in women with a trial of labor as compared to those who underwent ERCS [53]. However, when the analysis was restricted to low-risk women (women less than age 35, who attended at least one antenatal visit, with no pathology diagnosed during pregnancy, who were self-referred), the findings were no longer significant, leading the authors to conclude that low-risk women, by these criteria, had no increased risk of maternal complications or perinatal mortality for TOLAC as compared to ERCS [53].

## Patient opinion

Research from Tanzania on patient opinion regarding CS found that women preferred vaginal birth, they often reacted with fear and shock to their provider’s decision to move to CS, and perceived that there was a lack of indications for the procedure [54]. The study also found that religious beliefs and community members influenced patient’s opinions about CS, and that caregivers did not take the negative socioeconomic consequences of CS for the woman sufficiently into account in their decision-making [54]. In Nigeria, a structured questionnaire about CS was administered to women during antenatal care; only 4 felt that CS was ‘good’, 34 considered it ‘bad’ and would reluctantly undergo the procedure, while the remaining 225 (81.2 %) would accept CS if the life of their fetus was in danger; the authors conclude that this suggests that a significant proportion of antenatal clients were adverse to CS [55]. Another study from Nigeria found that 22 % of maternity clients actually refused CS, suggesting that sociocultural norms hindered acceptance of the procedure [56]. A study from Burkino Faso found that women are not only afraid of CS, but also feel guilty about the procedure afterwards, remarking that they were not good mothers because they could not give birth normally, and were concerned about the need for repeat CS in the future, the cost burden CS placed and would place on their families, and the risk of bad outcomes and poor quality of care [57].

As an interesting comparator, while most studies from Nigeria suggested that patients are not comfortable with CS a few articles from the same country documented the rise of cesarean delivery on maternal request (CDMR). In a study of just over 750 pregnant women, questionnaires were administered to evaluate women’s awareness of CDMR and willingness to request the procedure without the physician’s recommendation [58]. Author’s found that 6.4 % of patients reported willingness to request CS because they were motivated by fear of losing the fetus during labor and delivery, because they had had trouble conceiving, and because they were afraid of labor pains [58]. The analysis did not show any correlation between desiring CDMR and age, parity, or educational status [58]. Nigerian physicians, on the other hand, appear to have many patients requesting CDMR [59]. A study found that 94.4 % of consultants had had a patient request CDMR and 81.2 % of them had performed the procedure [59]. 88.9 % of the providers reported that is it important to accommodate the feelings of the women and offer CDMR out of respect for patient autonomy [59].

## Conclusions

This review shows that in SSA, the role of CS is complicated. Global health leadership (WHO) has asserted that the way forward in terms of safe labor and delivery practices that reduce adverse outcomes for mothers, fetuses, and babies, is that all women should deliver in a facility with a skilled birth attendant and access to comprehensive emergency obstetrical care [1]. This requires education of women, families, and communities and political will from providers, healthcare systems, and governments. It requires infrastructure, training, and most of all, money. But along with recommending facility delivery and the medicalization of childbirth comes a recognition of the fact that, providing quality care is the only way that obstetrics can tip the balance of the risks versus benefits of CS away from causing harm. It is up to obstetricians, their professional organizations, and global health leadership to establish evidence-based guidelines for the provision of safe CS in SSA and other LMIC. CS, the most commonly performed surgical procedure in the world, should be a high-quality life-saving technology that allows pregnant women, their offspring, and their support networks to continue to lead healthy, productive lives.

## Abbreviations

CDMR, cesarean delivery on maternal request; CS, cesarean section; EmOC, emergency obstetrical care; ERCS, elective repeat cesarean section; HIC, high-income country; IOL, induction of labor; LMIC, low- and middle-income countries; TOLAC, trial of labor after cesarean section; VBAC, vaginal birth after cesarean section
